# Marginal microleakage of flowable resin composites used to adhere Semi-Direct restorations

**DOI:** 10.1080/07853890.2021.1897362

**Published:** 2021-09-28

**Authors:** Inês de Sousa Correia, Inês Caetano Santos, Luís Proença, Mário Polido, Ana Azul

**Affiliations:** a Instituto Universitário Egas Moniz (IUEM), Egas Moniz Cooperativa de Ensino Superior, Caparica, Portugal; b Centro de Investigação Interdisciplinar Egas Moniz (CiiEM), Egas Moniz Cooperativa de Ensino Superior, Caparica, Portugal

## Abstract

**Introduction:**

Conservative Dentistry has gradually replaced traditional invasive procedures with minimally invasive techniques that rely on adhesion to tooth substrates [1]. Due to these recent developments, indirect restorations are increasingly used in contemporary practice. In order to bond these restorations, a vast choice of materials exist, and these include flowable resin composites [2,3]. However, studies featuring these materials are seldom found [3]. The aim of this *in vitro* pilot study was to evaluate and compare the microleakage of indirect restorations luted with a flowable composite and different luting agents.

**Materials and methods:**

This study was approved by the Ethics Committee of Egas Moniz, CRL. Thirty human molars were randomly divided between 3 groups (*n* = 10) according to the luting agent used: resin cement Bifix QM (VOCO GmbH, Cuxhaven, Germany) (G1), pre-heated resin composite Z100 MP Restorative (3 M ESPE) (G2) and flowable composite GrandioSO *Flow* (VOCO GmbH, Cuxhaven, Germany) (G3). Standardised class V cavities were prepared in the buccal surface and immediate dentine sealing was carried out with Optibond FL (Kerr). Composite restorations were made using a semi-direct technique with GrandioSO (VOCO GmbH, Cuxhaven, Germany). After 24 h cavities and restorations were pre-treated and adhesively luted according to the groups. After finishing and polishing, specimens were stored in distilled water at 37 °C for 24 h. After thermal aging (500 cycles at 5–55 °C) teeth were sealed and immersed in 0.5% basic fuchsine dye for 4 h. Each specimen was then sectioned vertically and microleakage was assessed and classified for both occlusal and cervical margins according to ISO/TS 11405:2015. The results were statistically analysed by Kruskal–Wallis (KW) test and binomial data analysis, at a significance level of 5% (SPSS 24.0).

**Results:**

There were no significant differences in microleakage scores between the tested groups (*p* > .05, KW). All the groups scored more leakage in cervical margins. Binomial analysis confirmed that the success rate was material and margin dependent. The probability of failure of a cervical margin bonded to Z100 (G2) and to GrandioSO Flow (G3) using a semi-direct technique was significantly higher than 50% (*p* = .021) and (*p* = .002) respectively. Z100 had 100% failure rate in the cervical margin.

**Discussion and conclusions:**

Longevity of a bonded interface is determined by sealing and microleakage. When this interface is compromised, failure of the restoration may happen [4]. Microleakage occurred in composite restorations made semi-directly the same way amongst the different materials used for bonding. However, cervical margins bonded with resin composites carry a greater chance of failure.
